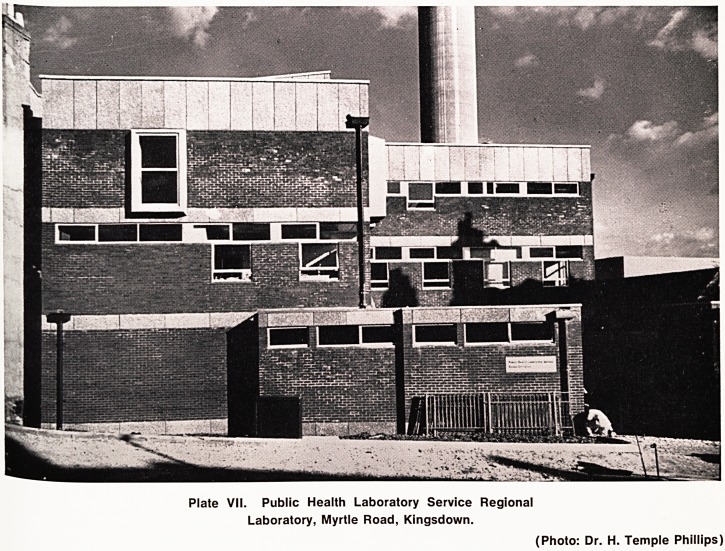# Canynge Hall 1933-1969

**Published:** 1970-01

**Authors:** R. C. Wofinden

**Affiliations:** Professor of Public Health, University of Bristol; Medical Officer of Health and Social Services, City and County of Bristol


### Canynge Hall, 1933-1969

### (A Short History of the University of Bristol Public Health Department)

by

**R. C. Wofinden, M.D., M.R.C.P., D.P.H., D.P.A.**

Professor of Public Health, University of Bristol; Medical Officer of Health and Social Services, City and County of Bristol.

Just over thirty-five years ago the newly formed University Department of Preventive Medicine was opened at Canynge Hall, a former hotel (Plate V), by the then Minister of Health, Sir Hilton Young. He described the occasion as the celebration of a marriage of the City of Bristol with the fair nymph of knowledge, and Dr. Stanley Badock, in proposing the vote of thanks, hoped that it would be a lasting and happy union.

By to-day's standards, the partners were strangely assorted—the Pathological Service provided by the University and the City's Chemical and Bacteriological Laboratories which were transferred from 36 Queen Square. Yet at that time, it was a logical step. Therapeutic medicine had few specific remedies to offer; communicable diseases were still a formidable cause of mortality and morbidity and the newly developing science of bacteriology was pointing the way to their prevention. Indeed, in the early 1930's the profession and the public had a healthier respect for preventive measures than in these days of ready access to a wide range of effective therapeutic agents. At that time there was also a need to provide the City's rapidly developing hospitals—at Southmead, Ham Green and Frenchay—with a bacteriological and pathological service. Preventive medicine was also being accorded more importance in the medical undergraduate curriculum.

The City Laboratories, under the direction of the Public Analyst, had hitherto dealt with the examination of diphtheria swabs and sputa; and samples of milk, water, food and drugs, rag flock, fertiliser and feeding stuffs. The University Department of Pathology had been responsible for the examination of specimens of fæces and urine from suspected cases of typhoid, paratyphoid and dysentery; blood and lochia, etc. from suspected cases of puerperal sepsis, syphilis and typhoid; and cerebro-spinal fluids. They also carried out animal inoculation tests for diphtheria and tuberculosis. Together, they now formed the new Preventive Medicine Department with the Medical Officer of Health (Dr. R. H. Parry), as the newly appointed Head and Professor of Preventive Medicine. Activities covered teaching, research and service, the latter not only to the City's Health Department and hospitals, but also to general medical practitioners. Dr. Walker Hall, who had resigned from the Chair of Pathology directed the combined laboratories for the first three years.

Meanwhile, the University Department of Pathology under Professor Geoffrey Hadfield, who succeeded Walker Hall in the Chair, became entirely academic, covering teaching and research in morbid anatomy and histology, and in bacteriology. It occupied the ground floor of Canynge Hall. From 1938 this Department became responsible also for a routine morbid anatomy service; during the war this included the conduct of post-mortems for all the Local Authority Hospitals as well as a part of this work for the teaching hospitals. After the war and the establishment of the National Health Service, the pathologists at Southmead and Frenchay undertook the morbid anatomy in their own laboratories, but the University Department at Canynge Hall continued to perform the post-mortems for the teaching hospitals. The Professor of Pathology was Curator of Canynge Hall and a very close association was maintained between the Departments of Preventive Medicine and Pathology and through them with all the hospitals in Bristol.

**40 Prince Street. The old Department of Public Health.**

From Dr. Hall's retirement in 1936 until 1947, direction of the Preventive Medicine Laboratories was carried out by Professor Parry. In those eleven years, there were important developments. Small laboratories were started at Southmead, Ham Green and Frenchay Hospitals; general practitioner demands for assistance increased and were met; the bacteriology teaching for medical students was transferred from the University Department of Pathology and a new bacteriology course was recognised as a subsidiary subject for the B.Sc., Technicians' training was also greatly improved.

In the reconstruction years immediately following the war further changes became necessary in the light of advances in scientific medicine and the introduction of a National Health Service.

Bacteriology had now "come of age" and due recognition was made by Reader status being conferred on Dr. K. E. Cooper (who had been appointed in 1938). He was promoted to Director of Laboratories which were also recognised by the Government as an Associated Laboratory of the National Public Health Laboratory Service. The bacteriologists and biochemists at Canynge Hall who had been serving the City's hospitals were now rehoused in expanded laboratories at Southmead, Frenchay and Ham Green. The bacteriologists remaining at Canynge Hall began to build up virological and immunological services and in 1951 Dr. Cooper was given Professorial status.

In 1946, Dr. W. H. Hobson was appointed full-time Lecturer in Preventive Medicine and Dr. G. Herdan as Statistician. A post-graduate Diploma Course in Public Health was re-introduced in order to help to make good some of the country's deficiency in public health doctors. The Health Visitors' Training Course was restarted at 36 Queen Square, the Public Health Inspectors' Course at the Technical College and the chemical laboratories at Canynge Hall were modernised and expanded.

Before the second World War the public health doctor had to be well-grounded in chemistry and bacteriology so that he could cope with the prevailing epidemic disease and the public health problems arising out of the physical environment. It was now clear that the second half of the century would require him to turn his attention to problems of the social environment and to the non-infectious, degenerative diseases which were assuming increasing importance.

Moreover, the creation of a national hospital service and a cadre of new consultants and specialists in tuberculosis and infectious diseases posed new problems in the career structure in the public health service. For a few years, General Medical Council Regulations permitted the organisation of a three months' Certificate Course in Public Health to precede the full Diploma in Public Health Course. It was designed for doctors proposing to work in sanatoria, infectious disease and psychiatric hospitals. It was quickly realised, however, that this was a mistaken conception and that such doctors were wiser to seek an M.R.C.P. which would help them to achieve consultant status and the C.P.H. was soon dropped.

As medicine became increasingly scientific, the need to provide a firm grounding in medical statistics and to teach public health doctors more about the principles and techniques of modern epidemiology led to the development in 1954 of a Joint Statistical Unit on a partnership basis by the City and the University. For convenience, it was housed at the Central Health Clinic and came under the immediate supervision of Dr. Herdan and Miss E. H. L. Duncan (the City's newly appointed Statistical Officer).

In 1955, Professor Parry retired, to be succeeded by Professor Wofinden in the following year, and the Department was renamed, the University Department of Public Health. In 1960, the chemistry laboratories were transferred back to the City under the direction of the Chief Scientific Officer (who is also the Public Analyst). In the same year, 21/23 Prince Street became the new home of the University Department of Public Health (Plate VI) providing accommodation for the Statistical Unit, the D.P.H. and the H.V. training courses. Dr. A. W. Macara was appointed Lecturer in Public Health in 1963.

Meanwhile, the new Medical School was nearing completion and the University Departments of Pathology and of Bacteriology were transferred to it in 1966. The vacant accommodation at Canynge Hall was then remodelled and now forms the new home of the University Department of Public Health. Dr. R. E. Midwinter has been appointed Lecturer in Epidemiology and Medical Statistics and Dr. P. N. Dixon as Lecturer in Public Health.

In 1969 the Public Health Laboratory Service Regional Laboratory which has been under the direction of Dr. H. R. Cayton since 1959 moved to a new building in St. Michael's Hill on the University-Teaching Hospital campus (Plate VII).

It is hoped that Canynge Hall will now be expanded into an epidemiological and medical statistical centre, with "on-line" connections to the University's Department of Computer Science.

The D.P.H. curriculum has been further revised under new General Medical Council Regulations. The course will form part of the vocational training of future medical administrators working in all branches of the National Health Service and of epidemiologists. The Health Visitors' Course has also been revised in accordance with the requirements of The Council for the Training of Health Visitors.

Medical undergraduates use Canynge Hall as their base while undertaking the one month's clerkship in Public Health and under their revised curriculum, it is hoped that Canynge Hall will also be the "base" for the teaching of "community medicine" in the third clinical year—a course which will involve many general medical practitioners and the City's health and social services.

With an augmented staff and the probable housing within the Department of the research unit of the Government's new council for health education, there are prospects of interesting and stimulating work in the next few years.

Over these last thirty-five years, academic public health in Bristol has had a somewhat chequered career, but there can be no doubt that Dr. Stanley Badock's hopes of a lasting and happy union between the City and the University have been fulfilled. At times the links have been somewhat tenuous, but on the whole it has been a fruitful union, facilitating the postgraduate training of many doctors and nurses working in all parts of the world and providing the opportunity for students from several university disciplines to study the living realities of health and social services at work. The senior staff of the City Public Health Department (most of whom have status as part-time lecturers in the University) have likewise benefited from the stimulating academic atmosphere in which they have worked.

In this brief historical report it has been impossible to pay tribute to all those who have played a part in

CLIFTON, BRISTOL.

### IMPERIAL HOTEL

**(Adjoining Clifton Down Station).**

| GENERAL TARIFF MODERATE. | EXCELLENT CUISINE. | ELECTRIC LIGHT THROUGHOUT. |
|---|---|---|

En Pension Terms from Two Guineas per week. Week-ends, 15/-.

**GOOD BILLIARD AND SMOKING ROOMS.**

**The "IMPERIAL"** is situated in the **most convenient position** for Visitors wishing to **see the beauties of Clifton and neighbourhood.** At the same time it is within easy reach of all the chief Places of Amusement. Electric Cars every minute to and from all parts of the City. Being on the direct Railway Route to Avonmouth, it is the best Hotel for all travellers by the **Jamaica and Canadian Mail Boats.**

Tel. No. 1555. Miss TEEK, Manageress.

Plate V. Canynge Hall—as it was.

these activities. Some of them are now dead, others are in retirement or are working overseas, a few are still in active service in the City. But all, by their efforts, have helped to demonstrate the benefits which can be gained from such a lasting partnership between the University Medical Faculty and the community in which it is situated.

**Acknowledgement**

My thanks are due to Emeritus Professor K. E. Cooper, Emeritus Professor T. F. Hewer, Dr. H. R. Cayton, Dr. H. T. Phillips and Dr. A. W. Macara for having read and checked this review.

**Plate VI. University Department of Public Health.**
**21-23 Prince Street.**

**(Photo: Dr. H. Temple Phillips)**

**Plate VII. Public Health Laboratory Service Regional Laboratory, Myrtle Road, Kingsdown.**

**(Photo: Dr. H. Temple Phillips)**

## Figures and Tables

**Figure f1:**
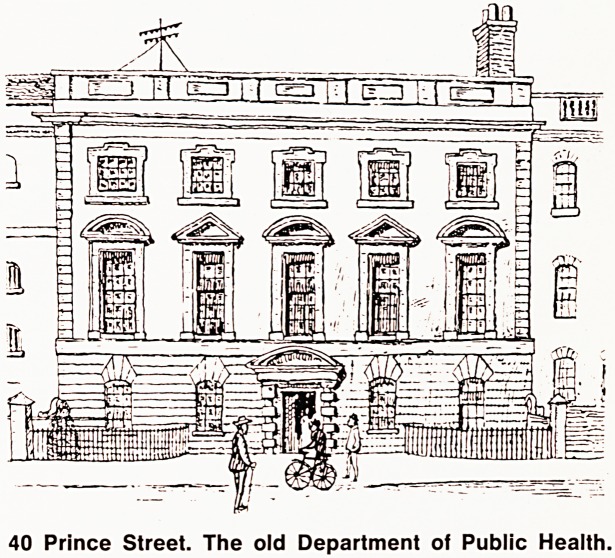


**Plate V. f2:**
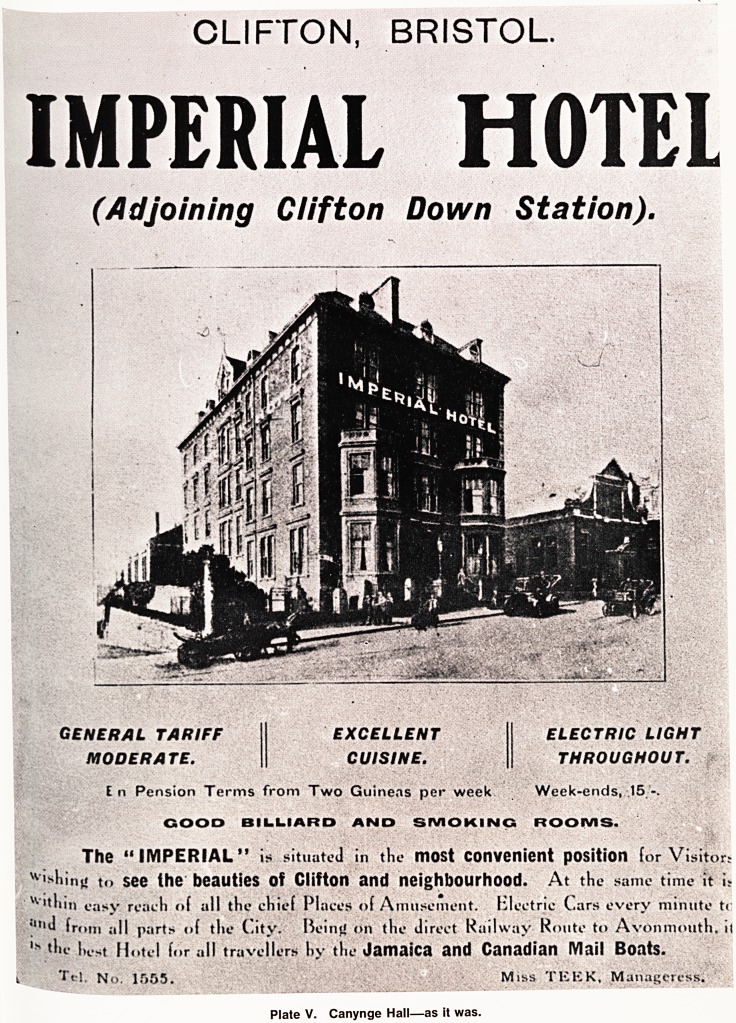


**Plate VI. f3:**
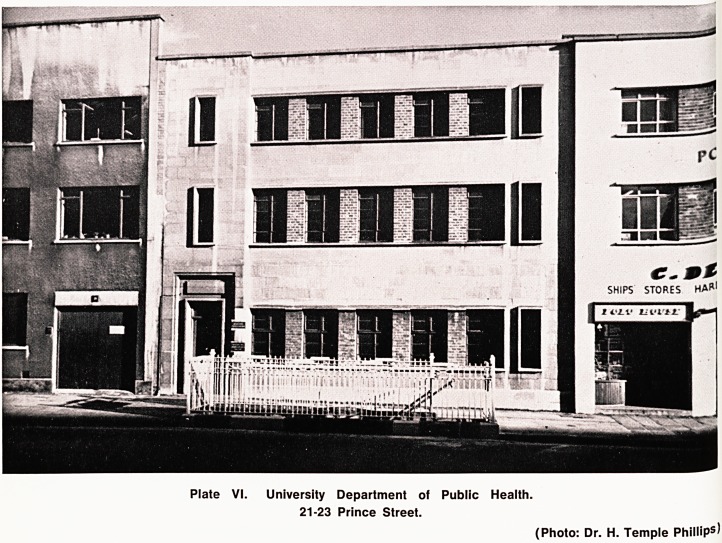


**Plate VII. f4:**